# Biofilm formation and antagonistic activity of *Lacticaseibacillus rhamnosus *(PTCC1712) and *Lactiplantibacillus plantarum *(PTCC1745)

**DOI:** 10.1186/s13568-021-01320-7

**Published:** 2021-11-25

**Authors:** Zeinab Rezaei, Saeid Khanzadi, Amir Salari

**Affiliations:** Department of Food Hygiene and Aquaculture, Faculty of Veterinary Medicine, Ferdowsi University of Mashhad, Mashhad, Iran

**Keywords:** Biofilm, Probiotics, Antagonistic activity, Food pathogen, CFS (cell-free supernatant), Postbiotic

## Abstract

Currently, the health benefits of probiotic bacteria are well known, and this has taken up a great deal of space in food science and health, both research and operational. On the other hand, anti-biofilm properties on food pathogens in the food and pharmaceutical industries have created an attractive challenge. This study aimed to describe the inhibitory activity of cell-free supernatants (CFS), planktonic cells, and biofilm form of lactobacilus strains (*L. rhamnosus and L. plantarum*) against food pathogens such as *Pseudomonas aeruginosa* and *Listeria monocytogenes*. Anti-bacterial activities of the CFS of lactobacillus strains were assessed by the microplate method and via violet staining. Evaluation of the antagonistic activity of planktonic cells and biofilm of LAB were performed by the spread plate method. The results showed the incubation time of 48 h was the best time to produce biofilm. Although the planktonic states reduce the pathogens bacterial about 1 –1.5 log, but in biofilm forms, decreased *L. monocytogenes* about 4.5 log compared to the control, and in the case of *P. aeruginosa*, a growth reduction of about 2.13 log was observed. Furthermore, biofilm formation of *L. monocytogenes* in the presence of *L. rhamnosus* cell-free supernatant was more weakly than *L. plantarum* CFS, but their CFS effect on reducing the bacterial population of *P. aeruginosa* was the same. According to the study, biofilm produced by probiotic strains can be considered a new approach for biological control. Also, cell-free supernatant can be used as postbiotic in the food and pharmaceutical industries.

## Introduction

Food safety is one of the most important issues for food producers and consumers. As food supply has become increasingly global, food safety issues need more attention (Moradi et al. 2020). For this purpose, a new approach of researchers and industries in recent years has been using biological policies in the face of the challenge of foodborne pathogens. Among the wide range of strategies currently being used or proposed, biocontrol based on organisms or their antimicrobial products has increased because of their popularity, no side effects, low processing costs, and low dependence on new technologies (Gálvez et al. 2010; Collazo Cordero et al. 2017). In the initial research, studies have focused on planktonic cells’ antimicrobial properties and their mode of action (McIntyre et al. 2007). However, recently, the use of free cells supernatant and biofilms of probiotics in biological control and development of intelligent antimicrobial surfaces are at the forefront of explorations (Guerrieri et al. 2009; Vuotto et al. 2014; Kaur et al. 2018). Biofilm is a membrane structure comprising a polysaccharide matrix, vitamins, proteins, and other components that surround microorganisms and have a complex internal structure and channels for transporting nutrients across the network. Nowadays, by discovering probiotics’ biofilm formation ability, this phenomenon has been introduced as a controller for pathogenic biofilms (Liu et al. 2015; Muhsin et al. 2015; Gómez et al. 2016; Speranza et al. 2020). Since this phenomenon is straightforward and inherent in bacteria itself, it can be used as an intelligent technique to control food pathogens (Balaure and Grumezescu 2020). Lactobacillus strains, which have the ability to auto-aggregation and co-aggregation, have antibiofilm properties. *L. rhamnosus* and *L. plantarum* are probiotic strains that have biofilm formation capacity and produced more robust biofilms than other species (Lebeer et al. 2007a, b; Simoes et al. 2009; Bujňáková and Kmeť 2012; Léonard et al. 2014).

Another version of probiotics is a cell-free supernatant, which in recent studies has been named post-biotic. This cell extract contains biosurfactant and antimicrobial agents produced during Lactobacillus growth and fermentation in complex growth conditions and which has been proposed as a method for biological control of pathogens (Abdelhamid et al. 2018; Chappell and Nair 2020). Since the *Listeria monocytogenes* (Leverentz et al. 2006; Warke et al. 2017; Kyere et al. 2020) and *Pseudomonas aeruginosa* are two food pathogens that have caused many problems in the food industry by producing biofilm (Rasamiravaka et al. 2015; Kumar 2016), therefore, in this study, antagonistic properties of three forms of planktonic, biofilm and cell-free supernatant of *L. plantarum* and *L. rhamnusus* were compared on these pathogens until to provides a better understanding of different forms of probiotic bacteria’s potency for their multifunctional use cluding food preservative agent, antibacterial and antibiofilm agent under different conditions.

## Materials and methods

### Lactobacillus and food patogene strains and culture conditions

Lyophilized culture of *Lacticaseibacillus rhamnosus* (PTCC1712) and *Lactiplantibacillus plantarum* (PTCC1745) isolated from pickled cabbage was obtained from the Iranian Research Organization for Science and Technology. The microbial culture was activated according to the company’s instructions. The activated bacteria were transferred into De Man, Rogosa, and Sharpe (MRS) broth or agar (Oxoid, Milan, Italy) and incubated under anaerobic conditions (Anaerobic conditions were achieved by the use of anaerobic jars with using Gas-Pack C.) at 37 °C for 72 h Kalantarmahdavi et al. (2021). *Listeria monocytogenes* (ATCC 7644) from American Type Culture Collection and *Pseudomonas aeruginosa* (PTCC 1074) were selected as food pathogens and cultured in Tryptic Soy broth (TSB, Oxoid).

### Probiotic biofilm formation assay

One milliliter of culture medium containing 1.5 × 10^8^ CFU mL^−1^ from each strain was poured into each well and incubated for 48 h at 30 °C. After incubation, the culture medium was drained from the wells and washed twice with 0.5 mL of 150 mM NaCl solution. The microplate was then stained for 45 min with 1 mL of 0.05% (v/v) of crystalline violet solution and washed twice. One mL of 96% ethanol (v/v) was added to each well, and the optical density was determined at 430 and 595 nm (Chen et al. 2017). To determine the best incubation time and in order to create a stronger biofilm, biofilm production was examined at intervals of 6, 12, 24, 36, 48, and 72 h of incubation.

### Antagonistic activity

The antagonistic activity of probiotic bacteria on food pathogens was investigated in three models: planktonic form, cell-free supernatant, and biofilm.

### Antagonistic activity of planktonic form

One milliliter of BHI (brain heart infusion) broth inoculated with 1.5 × 10^8^ CFU/mL of each pathogen strain was dispensed per well in a microplate. Then, One milliliter of fresh MRS broth inoculated with 1.5 × 10^8^ CFU/mL of lactobacillus strains was added. The microplate was incubated for 48 h at 30 °C. After incubation, the medium was removed from each well, and the microplates were washed twice with 500 mL of 150 mM NaCl solution. Evaluation of microorganisms was performed by the spread plate method. For each test, 1 mL of the samples was mixed with 9 mL of sterile peptone water. After sequential dilutions, appropriate dilutions were plated on set Oxford-Listeria-Selective-Agar (Base (Merck)) for *L. monocytogenes* and Pseudomonas agar base (Merck) for *P. aeruginosa.* Then, they were incubated at 37 °C for 72 h. The total counts of the viable bacteria were reported as logarithmic colony forming units per gram (log CFU/g). All the experiments were performed in triplicate, which means that each experiment was repeated at least three times.

### Antagonistic activity of LAB biofilms

Biofilm of LAB was formed in a microplate; then, one milliliter of fresh BHI broth inoculated with 1.5 × 10^8^ CFU/mL of *L. monocytognes* and *P. aeruginosa* was poured into wells that contained biofilm and incubated for 48 h at 30 °C. The number of *L.monocytogenes* and *P. aeruginosa* were counted by the spread plate method in selective media (Oxford-Listeria-Selective-Agar (Base) and Pseudomonas agar base, respectively). The control sample biofilm of the pathogen was formed similar to Lactobacillus biofilm (Zhang et al. 2013).

### Antibiofilm activity of the cell-free supernatant

To break down the membrane of a cell, 1.5 × 10^8^ CFU / mL of each lactic acid bacteria were subjected to sonication at 60 HZ for 5 min. Then, it was centrifuged (6000 *g*, 10 min, 4 °C), and supernatants were collected. 1.5 × 10^8^ CFU / mL of each pathogenic bacteria were inoculated into BHI broth, and 0.9 ml of it was poured into each well of a 24-well microplate, and 0.1 ml of supernatant was added to each well. Then, the microplates were incubated for 48 h at 37 °C, and to determine pathogen biofilm formation, OD value was measured at 430 and 595 nm (Kubota et al. 2008; Satpute et al. 2016). Adhesion rate was set to be B and can be calculated as followings: (Zhang et al. 2013; Chen et al. 2017)

$$B=\frac{{OD}_{B}-{OD}_{430}}{{OD}_{B}-{OD}_{595}}$$

OD_B_ refer to the optical density value in the Blank.

No biofilm producer = B<0.1;

Weak biofilm producer = 0.1≤ B <0.5;

Moderate biofilm producer = 0.1≤ B <1;

Strong biofilm producer = B ≥1.

### Investigation of biofilm microstructure by SEM

Biofilm was fixed in 2.5% glutardialdehyde solution in 10 Mm sodium cacodylate buffer for 24 h at 4 °C. It was then washed thrice for 15 min in 10 mM sodium cacodylate buffer by gentle mixing at room temperature, dehydrated in a graded ethanol series (50, 70, 80, 90, 95, and 100%). The samples were air-dried, placed on SEM stub, coated with gold/palladium by Sputter Coater device Model SC7620 (England), and investigated by a LEO1450VP scanning electron microscope (Germany) with resolution 2.5 nm and maximum voltage 35kv (Stefania et al. 2017).

### Statistical analysis

Data were analyzed by the one-way analysis of variance (ANOVA). If Onaway ANOVA was significant, the HolmeSidak test was used to determine significant differences (P < 0.05).

## Result

### The ability of biofilm formation by LAB over time

**Fig. 1:**
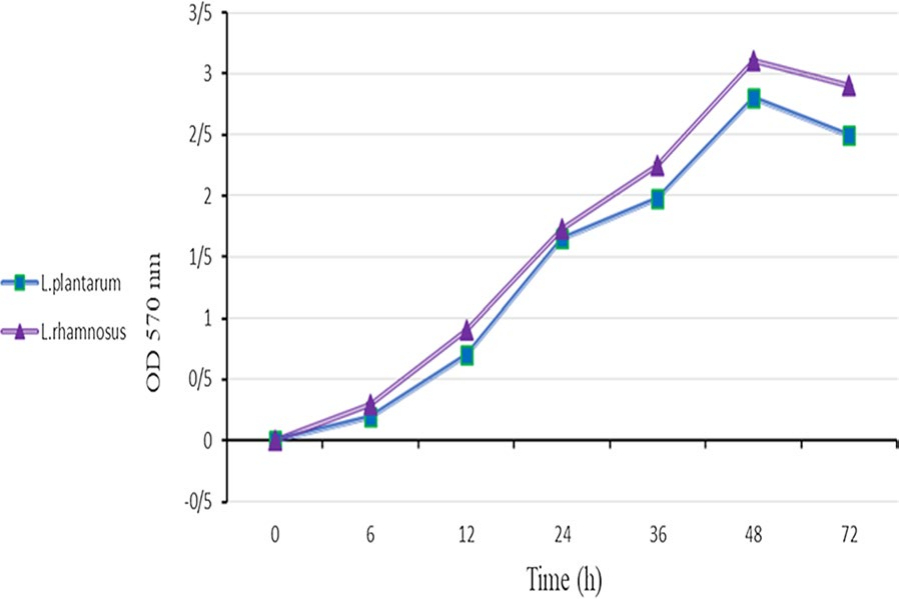
The ability of biofilm formation over time

The results showed that incubation time has a significant effect on biofilm formation. Figure 1 shows the process of biofilm formation over time; with increasing the incubation time, the rate of biofilm formation increased initially and reached its maximum value after 48 h. However, as incubation time continued, a decrease in biofilm formation rates in the strains was observed after 72 h. Notably, *L. rhamnosus* had a higher biofilm formation rate than *L. plantarum*.

### Antagonistic activity

#### Planktonic form

As shown in Table 1, the planktonic form of probiotics has reduced the growth of pathogens. Compared to the control sample, it has reduced pathogens’ growth by 1.1–1.5 log CFU/ml, but there is no significant difference in antagonistic properties between probiotic strains.

**Table 1:** Antagonistic activity associated with Lactobacillus strains on foodborne pathogen

Treatment		Food pathogen	
		*L. monocytogenes*	*P. aeruginosa*
Biofilm	*L. rhamnosus*	3.7 ± 0.1^c^	6.2 ± 0.7^b^
	*L. plantarum*	3.9 ± 0.01^c^	6.2 ± 2.3^b^
Planktonic cell	*L. rhamnosus*	6.7 ±0.3^b^	7.2± 0.5^a^
	*L. plantarum*	7.02± 0.5^b^	7.23 ± 0.11^a^
Control		8.3 ± 0.13^a^	8.3 ± 2.17^a^

Mean values in the same column followed by different superscript letters are significantly different (*P* < 0.05)

### Biofilm

The results demonstrated that the presence of *L. rhamnosus*, and *L. plantarum* biofilm, decreased *L. monocytogenes* by about 4.5 log compared to the control. In the case of *P. aeruginosa*, a growth reduction of about 2.13 log was observed. The obtained data show that the biofilm has more antagonistic power than the planktonic state; therefore, it has decreased 1.5 and 1 log CFU/ml in the case of *L*. *monocytogenes* and *P. aeruginosa*, respectively (Table 1).

### Cell-free supernatant antibiofilm activity

**Fig. 2:**
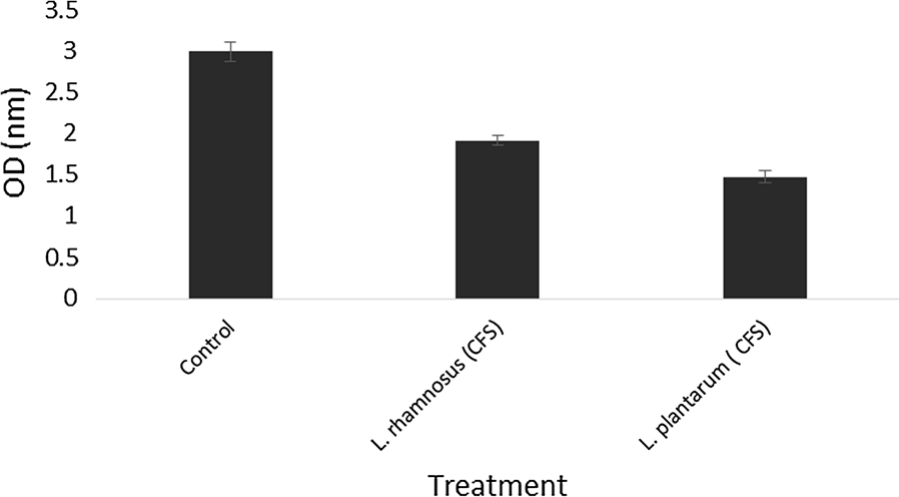
Antagonistic activity of the cell-free supernatant on *L. monocytogenes*

**Fig. 3:**
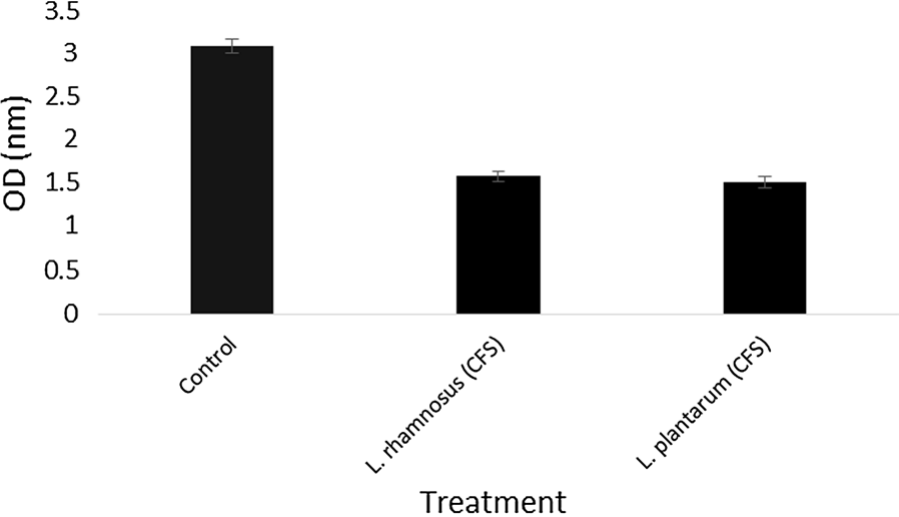
Antagonistic activity of the cell-free supernatant on *P. aeruginosa*

The results showed that the cell-free supernatants of probiotic bacteria had an effect on the biofilm formation of food pathogens and reduced their biofilm formation strength (Figs. 2 and 3). Meanwhile, in the presence of *L. rhamnosus* cell-free supernatant (CFS), the biofilm of *L. monocytogenes* formation was weaker than *L. plantarum* CFS, but their CFS effect reduces the bacterial population of *P. aeruginosa* was the same. Another point is that CFS L. *rhamnosus* had a more substantial inhibitory effect on the formation of *P. aeruginosa* biofilm. In general, the biofilms formed in the presence of CFS were much weaker than the control.

### Microstructure of biofilm

**Fig. 4:**
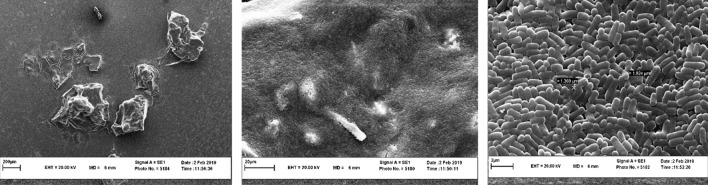
Scanning electron microscopy images of biofilm-forming *L. rhamnosus* in MRS

**Fig. 5:**
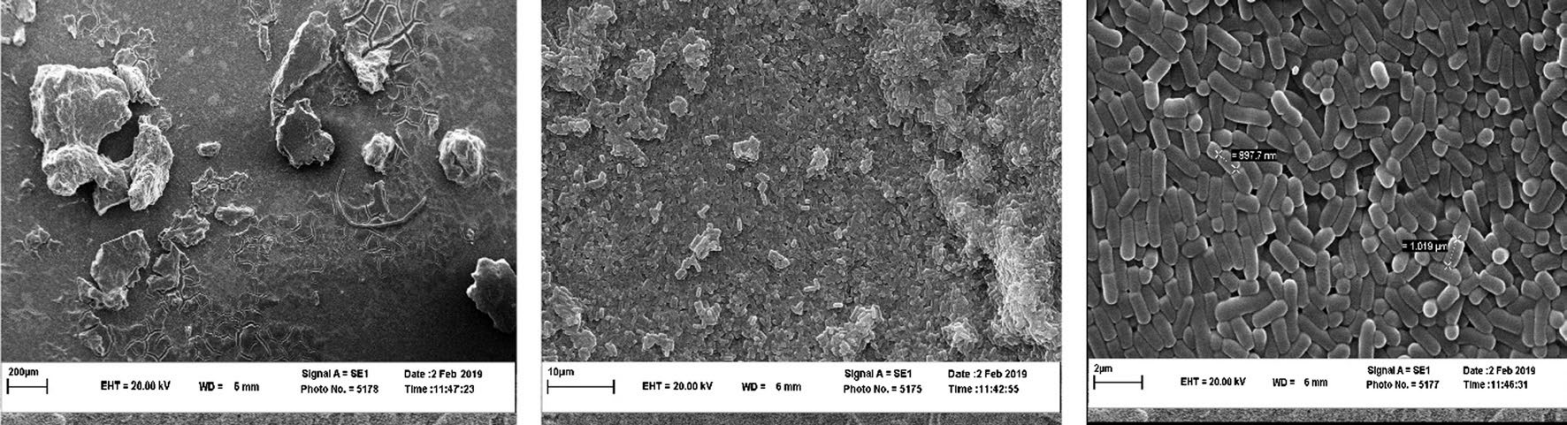
Scanning electron microscopy images of biofilm-forming *L. plantarum* in MRS

As can be seen from the comparison of the images (Figs. 4 and 5), the *L. rhamnosus* biofilm has a more uniform and impermeable surface than the *L. plantarum* and has a higher cell density, in general, it has formed a stronger biofilm, which is directly related to the biofilm formation power.

## Discussion

In this study, two strains of lactic acid bacteria were examined, and their potential for biofilm production was measured. Both strains were able to grow in the microplate and mature biofilm formation, and there was a slight difference in the biofilm density of the strains (Figs. 2, 3). In recent years, several studies have investigated the ability of *L. plantarum* and *L. rhamnosus* biofilm formation ability and their antagonistic activity in different forms separately.   Kaur et al. (2018) and Léonard et al. (2014) survey Planktonic cell of Lactobacillus strain on *Vibrio* and *L. monocytogenes* respectively. Also, Speranza et al. (2020) and Gómez et al. (2016) examinated the biofilm of probiotics on pathogens (Lebeer et al. 2007a, b; Guerrieri et al. 2009; Kubota et al. 2009; Bujňáková and Kmeť 2012; Vuotto et al. 2014; Muhsin et al. 2015). However, in most cases, a simultaneous comparison has not been performed. The results of this study showed that probiotic bacteria in all forms could have antimicrobial effects on pathogens, but this effect is more severe in biofilm form, which can be due to the nature of the biofilm and the number of bacteria and more antimicrobial compounds. Aoudia et al. (2016) showed that the biofilm supernatants of lactobacillus strains had more substantial effects than the supernatant produced by planktonic cells (Cotter et al. 2013). In a study by Satpute et al. (2016) the lactic acid bacteria showed high antimicrobial effects on the *L. monocytogenes*, which could be related to the presence of bacteriocin and biosurfactants compounds. Bacteriocins are antagonistic compounds that metabolic end-products are bactericidal proteins and substances similar to antibiotics (Stefania et al. 2017; Balaure and Grumezescu 2020). Other studies have shown that different strains of Lactobacillus isolated from dairy products were able to produce strong biofilms that can prevent the growth of food pathogens such as *Salmonella* and *E. coli* (Abdelhamid et al. 2018). Kubota et al. (2009) conducted studies on *L. plantarum*, examined bacterial resistance in both biofilm and planktonic states, and concluded that biofilm was effective in increasing bacterial resistance. Probiotics can produce bacteriocins, even in the face of intestinal infections (Cotter et al. 2013). Today, numerous studies have been performed in the field of bacterial therapy with probiotics, especially *L. plantarum* in human and animal models, which is not unrelated to these antagonistic compounds (Cotter et al. 2013; Argenta et al. 2016). Jones et al. (2012) reported that the polysaccharide compounds in biofilms act as TNF, a limiting factor, and exert their antagonistic effect (Jones et al. 2012). Since the bacterial population’s biofilm state is higher than the planktonic state, it has higher antagonistic potency. The investigation of the effects of time on biofilm formation showed that 48-h incubation time is the best time for strong and coherent biofilm formation. One of the most important achievements of the present study is the comprehensive investigation of probiotic bacteria’s antagonistic effects in their various forms. Analysis of the present study results showed that probiotics in three forms of planktonic, cell-free supernatant, and biofilm weaken pathogens’ growth. However, the bacterial antagonist’s simultaneous effect and the bacteriocin compounds produced and other antimicrobial compounds in the biofilm form are stronger and greater. Due to the fact that increasing resistance of foodborne pathogens compared to industrial disinfectants has created a serious challenge in the food and pharmaceutical industries and the environment. The biofilm of these bacteria at different surfaces and joints has created suitable growing conditions for them, endanger safety, quality, and stability. Therefore, researchers in recent years have investigated various competitive applications by probiotic bacteria, including natural antimicrobial products (Fijan et al. 2019; El-Mokhtar et al. 2020), bio factors and biofilm have been studied as a new way to control pathogenic bacteria (Jeong et al. 2018) and prevent food contamination. The second significant achievement of the present study is that if it was not possible to use probiotics as live bacteria, we could use their cell extracts as a natural preservative. in other applications can be used by forming biofilms on different surfaces of the industry to reduce the problem of biofilm formation of pathogens as a good idea to produce smart antimicrobial surfaces. Also, cell-free supernatant produced by probiotic strains which has recently been named Postbiotic can be considered a new generation of biological control agents and create a new approach in the food and pharmaceutical industries (Jiang et al. 2016; Tahmourespour et al. 2019; He et al. 2021).

## Data Availability

The corresponding author could provide all experimental data on a valid request.
